# Factors Associated with Non-Adherence to Drugs in Patients with Chronic Diseases Who Go to Pharmacies in Spain

**DOI:** 10.3390/ijerph18084308

**Published:** 2021-04-19

**Authors:** Carmen Valdés y Llorca, Ernesto Cortés Castell, José Manuel Ribera Casado, Pilar de Lucas Ramos, José Luis Casteig Ayestarán, Amaia Casteig Blanco, Vicente Francisco Gil Guillén, Mercedes Rizo Baeza

**Affiliations:** 1 Medical School, Miguel Hernández University, San Juan de Alicante, 03550 Alicante, Spain; cvaldesyllorca@gmail.com (C.V.yL.); josemanuel.ribera@salud.madrid.org (J.M.R.C.); pilar.delucas@gmail.com (P.d.L.R.); joseluis.casteig@fundoat.org (J.L.C.A.); amaia.casteig@oatobservatorio.com (A.C.B.); vte.gil@gmail.com (V.F.G.G.); rizo.mercedes@gmail.com (M.R.B.); 2 Scientific Committee Observatory Group Adherence to Treatment, 28231 Madrid, Spain; 3 Department of Pharmacology, Pediatrics and Organic Chemistry, Miguel Hernández University, San Juan de Alicante, 03550 Alicante, Spain; 4 Observatorio para la Adherencia al Tratamiento (OAT), 28231 Madrid, Spain; 5 Department of Clinical Medicine, Miguel Hernández University, San Juan de Alicante, 03550 Alicante, Spain; 6 Department of Nursing, University of Alicante, San Vicente del Raspeig, 03550 Alicante, Spain

**Keywords:** non-adherence, chronic diseases, pharmacological treatment, associated factors

## Abstract

Background. Pharmacological non-adherence in chronic diseases is 40–65%. No predictive profile of non-adherence exists in patients with multiple chronic diseases. Our study aimed to quantify the prevalence of non-adherence to pharmacological treatment and its associated factors in patients who visit pharmacies in Spain. Methods. This observational cross-sectional study included patients with one or more chronic diseases. The variables analyzed were demographics, diseases involved, self-medication, information about disease, and lifestyle. The main variable was adherence using the Morisky–Green test. A total of 132 pharmacies collaborated, providing 6327 patients representing all Spain regions (April–December 2016). Bivariate and multivariate analyses were performed and the area under the receiver operating characteristic (ROC) curve was calculated. Results. Non-adherence was 48.4% (95% confidence interval (CI): 47.2–49.7%). The variables that reached significance in the multivariate model were: difficulty in taking medication, self-medication, desire for more information, smoking, lower physical activity, younger age and number of chronic treatments. Discrimination was satisfactory (area under the ROC curve = 70%). Our study found that 50% patients was non-adherent and we obtained a profile of variables associated with therapeutic non-adherence. Conclusions. It is cause for concern that in patients with multiple diseases and taking multiple medications, there is an association between non-adherence, self-medication and worse lifestyle.

## 1. Introduction

Studies conducted in chronic diseases such as hypertension, diabetes mellitus (DM) and dyslipidaemia have determined the magnitude of pharmacological non-adherence to be 40–65% [1,2,3,4]. With regard to the profiles of variables associated with non-adherence, our working group in Spain concluded years ago that no prototype exists of the non-adherent patient [5,6,7,8]. However, traditionally, four main factors have been associated with poor treatment adherence: those associated with the dependent variables of the individuals, such as sex, age, cultural level, economic level, and personal beliefs; those associated with the physician–patient relationship, due to the variability among health professionals in empathy, motivational clinical interviewing, effective communication and shared decision-making; those associated with the work and family environment of the patient; and those associated with therapeutic complexity [5,6,7,8,9,10,11,12,13,14,15,16].

In other studies, the most consistent factors found to be associated with greater pharmacological non-adherence are those dependent on therapeutic complexity, such that patients with multiple diseases and multiple medications have a higher probability of non-adherence to pharmacological treatment [17,18,19,20,21,22,23,24,25]. Few studies use a holistic approach to address therapeutic adherence in chronic patients, as most studies focus on specific chronic diseases such as hypertension, DM, dyslipidaemia, depression or osteoporosis [5,6,7,8,9,17,18,19,20,21,22,23,24,25].

In Spain, community pharmacies are ideal places not only to measure and investigate therapeutic non-adherence, but also to carry out interventions to help reduce pharmacological non-adherence in chronic patients [23,24,25,26]. The majority of studies undertaken on therapeutic adherence have been conducted in primary care or specialized care [5,6,7,8,17,18,19,20,21,22,23,24,25], but fewer studies are conducted in pharmacies [27,28,29].

The aim of this study was to quantify adherence to pharmacological treatment and to assess the factors associated with non-adherence in patients with multiple chronic conditions who go to Spanish pharmacies for their drugs. The importance of the information resulting from this study lies in establishing strategies based on these factors to achieve greater therapeutic adherence. 

## 2. Methods

This observational cross-sectional study involved all the Spanish Autonomous Communities, with the exception of the autonomous cities of Ceuta and Melilla. As inclusion criteria, patients were selected who visited Spanish community pharmacies to collect the medication prescribed by their physician at their respective health centers and who had a diagnosis of at least one chronic disease, such as DM, hypertension, chronic obstructive pulmonary disease, asthma, hypercholesterolaemia, rheumatic disease, depression, or overactive bladder, which required prolonged pharmacological treatment (life-long medication). The exclusion criterion was the unwillingness to respond to the questionnaire provided. The pharmacist invited the patient to participate in the study, and the participation level was nearly 80%, which is appropriate for this type of study. The data were obtained by direct interview between the patient and the pharmacist and lasted an average of 10 min.

### 2.1. Study Variables

The specially-designed survey contained five main sections: (i) Personal data, with anthropometric and socioeconomic variables (age, gender, height, weight, educational status, income and living status). (ii) Conditions the patient has and the drugs used. The data obtained provide information about the chronic disease or diseases that the patient has and whether he or she is being treated (DM, hypertension, chronic obstructive pulmonary disease, asthma, high cholesterol, osteoporosis, rheumatic diseases, depression, heart disease, overactive bladder, or other chronic conditions). (iii) Adherence, through a survey constructed based on the Morisky–Green test, to determine the level of treatment adherence [30] (difficulty in taking the tablets every day through self-reported adherence, self-medication and whether the patient uses a mnemonic trick to help remember to take the medication). (iv) Information about the disease (the patient would like to have more information about the disease/treatment and has a caregiver). (v) Lifestyle and healthy habits (to learn whether the patient is a smoker, is physically active in his or her free time and whether he or she is currently following a special diet or dietary regimen). 

The main variable was the measurement of adherence according to the Morisky–Green test [30], considering non-adherence to be present when the patient did not respond appropriately to one of the four test questions: Answered yes to the question “Do you ever forget to take your medication?”; no to “Do you take your medication at the indicated time?”; yes to “When you feel good, do you ever stop taking it?”; or yes “If you feel bad, do you ever stop taking your medication?” The rest of the variables were analyzed as independent variables. 

The survey was adapted to enable automatic reading of the data and to proceed with the most reliable recording method. Convenience sampling was undertaken to identify pharmacies and stratified by proportional allocation, using as a reference the distribution of the Spanish population by province. A total of 132 community pharmacies collaborated. The patients were recruited consecutively according to the order of arrival at the pharmacy and were administered the survey following oral consent after the study was explained to them. The fieldwork was carried out from the second week of April 2016 until December 2016, with a break during the summer months for holidays. To calculate the sample size, the Spanish population figure from the National Institute of Statistics consulted on 01 January 2016 was used as a reference. The confidence level was set at 99.9% with a ±2% sampling error, and the maximum variability of the parameter p × q = 0.25. With these assumptions, the sample obtained was 6006 subjects.

### 2.2. Statistical Analysis

The statistical analysis was performed according to the type of variable. Qualitative variables are shown as their absolute and relative frequencies and quantitative variables as the mean and standard deviation. For the bivariate analysis, proportions were analyzed with the Chi-square test and means compared with Student’s *t*-test. In order to minimize confounding bias, a multivariate analysis was carried out using binary logistic regression, using therapeutic non-adherence as a dependent variable as per the Morisky–Green test. The odds ratios were calculated with their 95% confidence interval (CI). To evaluate the discriminatory ability of the model, the area under the receiver operating characteristic (ROC) curve and its 95% CI were calculated. The calibration method used was the Hosmer–Lemeshow test. All statistical analyses were performed with the statistical package SPSS version 24.0 (IBM Corp., Armonk, NY, USA).

### 2.3. Ethical Considerations

Approval for the study was given by the Clinical Research Ethics Committee of the Spanish Agency of Medicines and Medical Devices (Ref. OBS-MED-2015-01) as well as by the Ethics Committee of the Clinical Hospital of Madrid (Ref. C.I. 15/251-E). The study abided by good clinical practice guidelines. The data collection sheet contained no patient identification, and the analysis was encrypted and undertaken anonymously.

## 3. Results

The results of the descriptive, bivariate and multivariate analysis are shown in Table 1. 

The goodness of fit of the regression model was determined using the Hosmer–Lemeshow test: *p* = 0.420. The area under the ROC curve was used for discrimination: 0.70 (CI 95%: 0.68–0.71, *p* < 0.001). 

The second column of the table presents the descriptive analysis of the sample. Of note is that 56.7% were women, and the mean age was 64.7 ± 15.9 years. The predominant educational level was primary/others, at 50.5%. Regarding income, 24.9% had an income level of 800–1300 euros/month, followed by 21.3% with an income level <800 euros/month (the Spanish minimum wage in 2016 was 655.20 euros/month, although this has now risen to 858.55 euros a month). Eighteen per cent of the patients lived alone, 35.2% self-medicated, 17.4% had a caregiver, 43.9% desired more information on treatments and adherence, and 18% currently smoked. Concerning physical activity, 45.7% of the patients were not physically active. The analysis of chronic diseases revealed that 54.7% of the patients had hypertension, 49.1% had dyslipidaemia, 29.9% had DM, and 24.4% had depression. A total of 75.3% had two or more chronic diseases and 71.9% took two or more daily treatments. The mean number of chronic diseases and chronic treatments in the sample was 2.5 ± 1.5 and 2.3 ± 1.3, respectively. The mean body mass index was 27.3 ± 4.9 kg/m^2^. 

The Morisky–Green test showed a non-adherence rate of 48.4% (95% CI: 47.2–49.7%). The third and fourth columns of Table 1 describe the bivariate analysis of treatment non-adherence. The profile of variables associated with non-adherence comprised: difficulty with medication (*p* < 0.001), self-medication (*p* < 0.001), having a caregiver (*p* = 0.023), wishing for further information about their disease (*p* < 0.001), smoking (*p* < 0.001), a lower level of physical activity (*p* < 0.001), not following a diet (*p* = 0.020), younger age (*p* < 0.001), a higher number of chronic diseases (*p* = 0.047) and a higher number of chronic treatments (*p* = 0.001).

The fifth and sixth columns of Table 1 display the multivariate analysis by logistic regression. The goodness of fit of the regression model through the Hosmer–Lemeshow test did not reach significance (*p* = 0.420), indicating that the model has a good fit between the observed and expected non-adherent patients. In addition, the discrimination was satisfactory, with an area under the ROC curve of 70%. The variables associated with greater non-adherence in the multivariate analysis and which reached statistical significance were: having difficulty taking the medication (*p* < 0.001), self-medication (*p* < 0.001), wishing for further information (*p* < 0.001), smoking (*p* = 0.025), a lower level of physical activity (category none, *p* = 0.006 and high *p* < 0.001), a lower age (*p* = 0.001) and needing two to three (*p* = 0.015) and four or more (*p* < 0.001) chronic treatments.

## 4. Discussion

Studies on adherence in chronic diseases have been carried out primarily in specific diseases, the most prevalent being hypertension, DM and dyslipidaemia [1,2,3,4,5,6,7,8,9,10,11]. In these studies, the magnitude of pharmacological non-adherence is around 50%. In clinical practice, the impact of non-adherence is that when patients fail to take the medication properly, the benefits of the treatments are not achieved.

Our results demonstrate that in Spanish chronic patients, where approximately three out of four have more than one disease and chronic treatments, about half have poor treatment adherence. This non-adherence can generate a troubling lack of control in complex patients, with a mean age of approximately 65 years, who have multiple diseases and take multiple medications and where the most prevalent chronic diseases in our care setting coexist, including hypertension, DM, dyslipidaemia, and depression.

In the study of the profile of factors associated with non-adherence, seven variables reached statistical significance, generating a multivariate model with an area under the ROC curve (AUC = 0.7) indicating acceptable discriminatory ability to identify patient non-adherence in the clinical care setting. From the individualized analysis of each of these variables, based on their measures of association according to the odds ratios obtained (Table 1), we will be able to design strategies focused on reducing non-adherence in these complex patients to improve control of their chronic diseases. Examination of other studies found no consistency regarding the relationship between non-adherence and dependent socioeconomic, demographic and work-family factors [5,6,7,8,9,10,11,12,13,14,15,16], although all the authors agree that non-adherence is associated with greater therapeutic complexity and a poorer doctor-patient relationship [17,18,19,20,21,22,23,24,25].

The main factor associated with non-adherence in our study was recognition by the patients that it was difficult for them to take their medication every day, as described by those patients in response to the question on the self-report survey on adherence. 

This response, therefore, indicates that the probability of non-adherence is more than five times greater in these patients (odds ratio (OR) = 5.2). As in other studies, this result is explained by the high specificity obtained when this method is validated [31,32,33,34]. The issue is that only one out of every six patients in our study responded that they had difficulty taking their medication. 

In our study, we found a relationship between those patients who recognized that they were self-medicating and non-adherence. Patients who self-medicated were almost twice as likely to be non-adherent (OR = 1.9). Other studies should corroborate this result. 

The association between non-adherence and poorer habits in the chronic population is cause for concern. We found that patients were less likely to be adherent if they were smokers (OR = 1.2) or were less physically active (OR 1.3–1.6, depending on the degree of physical exercise) had higher non-adherence. These patients must be identified in order to avoid the consequences, not only of the poor control of their diseases due to non-adherence, but also because they have a higher cardiovascular, tumor and frailty risk due to smoking and a more sedentary lifestyle.

Our analysis has shown that patients who wish to have more information about their conditions and chronic treatments are non-adherent almost one and a half times as often (OR = 1.4). This result indicates that to overcome non-adherence in clinical practice, health professionals should promote and prioritize educational techniques to motivate patients and which incorporate better knowledge about chronic diseases. The reasons for adhering to chronic treatments should also be explained.

The association between greater therapeutic complexity and non-adherence in our study corroborates the association found by most authors that the greater the number of tablets, the greater the likelihood of non-adherence [19,20,21,22]. In our study, the highest percentages of non-adherence occurred when the patient took four or more tablets daily (OR = 1.6). Controversy exists regarding the association between age and treatment adherence [5,6,7,8,9,10,11,12,13,14,15,16]. Some studies have found greater non-adherence in older patients due to forgetfulness, although others have failed to find this association [5,6,7,8,9,10,11,12,13,14,15,16]. However, in our patients non-adherence was higher in the youngest patients. More studies are needed to confirm these results in patients with several chronic diseases who take more than one chronic treatment.

The major strengths of this work are the methodology used and the clinical question answered. The inclusion of more than 6000 patients selected through a sample stratified by autonomous communities enabled us to generalize our results and minimize random error. Additionally, rather than studying treatment adherence in specific diseases, we studied adherence in chronic patients, many of whom had multiple diseases and were taking multiple medications. Therefore, the magnitude found corresponds more closely with clinical practice. In addition, most adherence studies have been conducted in primary care centers and hospitals [4,5,6,7,21,22,23,27], but very few in community pharmacies [24,25,26]. Our results indicate that this setting is particularly appropriate for addressing issues related to therapeutic non-adherence in complex chronic patients, and the participation of healthcare professionals should be encouraged to overcome this important health problem. 

The limitations are due to the cross-sectional design in which adherence was measured only once; therefore, causality could not be established. In view of the results of this study, our working group will undertake further research using a longitudinal analytical design to assess changes and causality. With respect to biases, in any adherence study, measurement bias is accepted since due to its complexity no ideal method exists. However, the Morisky–Green test that we used has been validated in Spain [3,31,32,33] and has been used in relevant studies [34,35]. Concerning selection bias, it was assumed that the population visiting community pharmacies were patients collecting their own prescriptions. Thus, those patients who for reasons of work or disability-mobility did not go to collect their medication would be missing. Furthermore, approximately 20% of the patients refused to participate in the study, although this is a low percentage in this type of study, and we believe this was due to the use of a direct interview with the pharmacist, which involved a delay of about 10 min [23]. To minimize confusion bias, we performed a multivariate analysis.

We can conclude that one out of two persons with chronic diseases who go to community pharmacies for their drugs fail to adhere to pharmacological treatment. Questioning the patients using the self-report method is the strongest predictor for identifying non-adherent patients. The patient profile we have obtained has adequate discriminatory ability and is useful for prioritizing interventions aimed at reducing non-adherence to pharmacological therapy. In individuals with several chronic conditions and various different medications the association between non-adherence to pharmacological treatment and self-medication and poorer lifestyles is a cause for concern and should be corroborated by other studies.

## 5. Conclusions

* Studies in patients with chronic diseases find that 40–65% are non-adherent to drug treatment.

* There is no predictive profile of variables associated with the lack of adherence to treatment.

* Traditionally, four main factors have been associated with poor adherence related to the social, economic and cultural characteristics of the patient, the doctor–patient relationship, the work and family environment of the patient, and the therapeutic complexity.

* Pharmacy offices are ideal places to help primary care teams in the control of chronic pathologies through the identification of non-compliant patients.

* Questioning the patients using the self-report method is the strongest predictor for identifying non-adherent patients.

* Non-adherence to drug treatment in patients with multiple chronic diseases and multiple medications is associated with self-medication and unhealthy lifestyles.

## Figures and Tables

**Table 1:** Analysis of pharmacological non-adherence in a representative sample of the Spanish population with chronic diseases.

Variable	Total *n* = 6327 *n* (%) */x ± s	Non-Adherence *n* = 3065 (48.4%) *n* (%) **/x ± s	*p*	AOR (95% CI)	*p*
Female sex	3585 (56.7)	1737 (48.5)	0.959	0.94 (0.83–1.07)	0.381
Education:					
Primary/other	3193 (50.5)	1528 (47.9)	0.484	N/I	N/I
High school	996 (15.7)	498 (50.0)			
Vocational training	562 (8.9)	279 (49.6)			
University	1404 (22.2)	663 (47.2)			
Monthly income (€):					
<800	1347 (21.3)	663 (49.2)	0.250	N/I	N/I
800–1300	1574 (24.9)	793 (50.4)			
1300–1850	954 (15.1)	445 (46.6)			
1850–2700	456 (7.2)	210 (46.1)			
2700–3450	201 (3.2)	107 (53.2)			
>3450	89 (1.4)	46 (51.7)			
Lives alone	1140 (18.0)	579 (50.8)	0.090	1.02 (0.88–1.19)	0.776
Difficulty taking medication	1051 (16.6)	852 (81.1)	<0.001	5.18 (4.26–6.31)	<0.001
Mnemonic trick	1746 (27.6)	826 (47.3)	0.438	N/I	N/I
Self-medication	2225 (35.2)	1331 (59.8)	<0.001	1.86 (1.62–2.12)	<0.001
Has caregiver	1098 (17.4)	562 (51.2)	0.023	1.10 (0.93–1.31)	0.265
Desires more information	2776 (43.9)	1470 (53.0)	<0.001	1.42 (1.25–1.62)	<0.001
Current smoker	1140 (18.0)	647 (56.8)	<0.001	1.22 (1.03–1.45)	0.025
Physical activity:					
None	2893 (45.7)	1506 (52.1)	<0.001	1	
Low	1571 (24.8)	753 (47.9)		0.80 (0.68–0.94)	0.006
Average	745 (11.8)	362 (48.6)		0.88 (0.71–1.08)	0.225
High	1038 (16.4)	396 (38.2)		0.63 (0.52–0.76)	<0.001
Follows a diet	2236 (35.3)	1034 (46.2)	0.020	0.90 (0.79–1.03)	0.138
Age (years)	64.7 ± 15.9	63.7 ± 16.3	<0.001	0.99 (0.99–1.00)	0.001
Number of chronic diseases:					
1	1566 (24.8)	722 (46.1)	0.047	N/I	N/I
2–3	3011 (47.6)	1461 (48.5)			
≥4	1750 (27.7)	882 (50.4)			
Number of chronic treatments:					
1	1776 (28.1)	838 (47.2)	0.001	1	
2–3	3289 (52.0)	1555 (47.3)		1.22 (1.04–1.43)	0.015
≥4	1262 (19.9)	672 (53.2)		1.57 (1.29–1.92)	<0.001
BMI (kg/m^2^)	27.3 ± 4.9	27.3 ± 4.9	0.798	N/I	N/I

Abbreviations: *n* (%), absolute frequency (relative frequency); N/I, not included in the model; AOR, adjusted odds ratio; x ± s, mean standard deviation; BMI, body mass index. * Percentage of the total sample; ** Percentage of non-adherent patients in each of the variables analyzed.

## Data Availability

The data are available on a reasoned request by contacting the reference author.
